# Sensemaking, adaptation and agency in human-exoskeleton synchrony

**DOI:** 10.3389/frobt.2023.1207052

**Published:** 2023-10-12

**Authors:** J. Nan Wilkenfeld, Sunwook Kim, Satyajit Upasani, Gavin Lawrence Kirkwood, Norah E. Dunbar, Divya Srinivasan

**Affiliations:** ^1^ Department of Communication, University of California, Santa Barbara, Santa Barbara, CA, United States; ^2^ Industrial and Systems Engineering Department, Virginia Tech, Blacksburg, VA, United States; ^3^ Department of BioEngineering, Clemson University, Clemson, SC, United States

**Keywords:** exoskeletons, wearable robots, coadaptation, collaborative robotics, HRI (human robot interaction), anthropomorphization

## Abstract

**Introduction:** Wearable I robots such as exoskeletons combine the strength and precision of intelligent machines with the adaptability and creativity of human beings. Exoskeletons are unique in that humans interact with the technologies on both a physical and cognitive level, and as such, involve a complex, interdependent relationship between humans and robots. The aim of this paper was to explore the concepts of agency and adaptation as they relate to human-machine synchrony, as human users learned to operate a complex whole-body powered exoskeleton.

**Methods:** Qualitative interviews were conducted with participants over multiple sessions in which they performed a range of basic functional tasks and simulated industrial tasks using a powered exoskeleton prototype, to understand their expectations of the human-technology partnership, any challenges that arose in their interaction with the device, and what strategies they used to resolve such challenges.

**Results:** Analysis of the data revealed two overarching themes: 1) Participants faced physical, cognitive, and affective challenges to synchronizing with the exoskeleton; and 2) they engaged in sensemaking strategies such as drawing analogies with known prior experiences and anthropomorphized the exoskeleton as a partner entity in order to adapt and address challenges.

**Discussion:** This research is an important first step to understanding how humans make sense of and adapt to a powerful and complex wearable robot with which they must synchronize in order to perform tasks. Implications for our understanding of human and machine agency as well as bidirectional coadaptation principles are discussed.

## Introduction

The past decade has seen an explosion in the development and usage of intelligent machines working collaboratively with human employees across a wide variety of industries. These machines are designed with the goal of augmenting human performance, rather than replacing them, thereby improving overall industrial productivity while maintaining the creativity and flexibility of human employees. A significant area in collaborative industrial robotics is the development of wearable robots called exoskeletons (Kazerooni, 2005; Ansari et al., 2015; De Looze et al., 2016; Bogue, 2018). “Industrial exoskeletons” is the collective name given to mechanical devices worn by workers, whose construction mirrors the structure of the operator’s limbs, and is utilized as an amplifier of human strength or as a fatigue/strain reducer (Lowe et al., 2019). Body weight support, lift assistance, load maintenance, positioning correction and body stabilization are common capabilities of industrial exoskeletons (de Looze et al., 2016; McFarland and Fischer, 2019).

Exoskeletons are being developed for use in industrial applications to reduce musculoskeletal injuries, worker strain, and fatigue from performing repetitive and laborious tasks (de Looze et al., 2016; Lowe et al., 2019; Theurel and Desbrosses, 2019). These technologies represent a unique type of wearable technology because, not only are humans interacting with these machines on both a physical and cognitive level, the human and machine are physically and cognitively interdependent, which necessitates a high level of collaboration and coordination (Pons, 2008; Ronsse et al., 2011). On the one hand, exoskeletons are used to support and increase the strength and mobility of the wearer (Bequette, et at., 2020; Stirling et al., 2020; Zhu et al., 2021) and on the other, human cognition including adaptability is used to support the work of the robot (Pons, 2008). This interdependence is an example of the increasingly egalitarian relationships between humans and machines, and how they need to synchronize to become one functioning entity.

Humans attempt to coordinate their behaviors, or synchronize, with non-human agents such as computers and robots, as they do with other humans (Fujiwara et al., 2022). This drive to synchronize is increased when human-robot teams are tasked with accomplishing a common goal. Much of prior research on synchrony with non-human agents has focused on performance or affective outcomes of synchrony. For example, Fujiwara et al. (2022) found that synchrony behaviors with non-human avatars in a collaborative task resulted in people having favorable impressions of the avatar and more advantageous outcomes in a negotiation. Studies like this have primarily used a unidirectional perspective such as the machine adapting to the user, or the user reacting to the machine. However, Burgoon et al. (1995) argue that adaptation is often bi-directional, and is, in reality, “co-adaptation.” Sometimes one partner may adjust to the other (unidirectional), and at other times the influence may be mutual or perhaps even alternate between the two partners in who takes a leadership role.

Exoskeleton technologies provide a unique opportunity for researchers to understand bi-directional adaptation between humans and machines as they use “shared control” (Ronsse, 2011, p. 1001), to achieve a common task goal that both the human user and exoskeleton contribute towards, such as lifting a heavy object. Ideally, each agent needs to adapt to the other to synchronize and complete the task: the human adjusts their movements to potentially leverage the strengths of the exoskeleton, and the robot is typically designed to respond appropriately to human movement (and intent) by adjusting its torque and kinematics. However, while several attempts have been made to improve the synchrony between humans and robots from a robot controls/design perspective, (e.g., Losey et al., 2018), there are relatively unexplored factors related to human agency and preferences that may disrupt the synchrony between humans and wearable robots. When conceptualizing factors that would impact a wearer’s ability to synchronize with an exoskeleton, feelings of wearer agency, task goals, and the norms of technology use in a work environment have been suggested as factors that would likely determine the success of the co-dependent relationship (Kirkwood et al. (2021).

In defining synchrony, interaction coordination is an intrinsic component of human behavior and can be considered the fundamental building block upon which all relationships rely (Berneri and Rosenthal, 1991; Burgoon et al., 1995). Interactional synchrony, a type of interaction coordination, is generally defined as the degree to which separate, endogenous behavioral patterns match, and is comprised of three components: rhythm, simultaneous movement, and the smooth meshing of interaction (Berneri and Rosenthal, 1991. P. 403). While much early work on synchrony explored body postural similarities in human dyads, technological advancements have enabled researchers to explore additional rhythm and timing components, which may more deeply elucidate synchrony (referred to as “essence of synchrony” by Fujiwara and Daibo, 2016). Oft cited examples of the importance of these components are jazz bands and sports teams whose members may differ in their posture and movements but synchronize through rhythm and timing to form a larger, singular entity (Bernieri and Rosenthal, 1991; Lorenz et al., 2013; Fujiwara and Daibo, 2016). In the context of human-machine synchrony, empirical work has shown that machines that move, and particularly machines that move synchronously with their human-counterparts, are perceived as more “likeable” and “intelligent” by humans (Lehmann et al., 2015).

Humans prefer reciprocity and synchrony as part of their “required” human needs and drives related to their survival, safety, comfort, and affiliation according to Interaction Adaptation Theory (IAT; Burgoon et al., 1995), and this need or desire for connection extends to their non-human interaction partners (Lehmann et al., 2015). Though there is as of yet little research on synchrony between humans and machines, related studies have provided empirical evidence that humans will make an effort to build rapport and coordinate movements with non-human agents and receive the affective and performance benefits that come from this synchronization (Nikolaidis et al., 2016; Nikolaidis et al., 2017; Fujiwara et al., 2022). Most prior work on human-robot synchrony has particularly focused on the temporal/rhythmic coordination aspects of synchrony: for example, children were found to adapt the timing of their movement behaviors (in an interactive drumming task) to match that of a humanoid robot’s behavior (Robins et al., 2008), and bidirectional temporal motor coordination patterns were reported between humans and a variety of mobile robots Dautenhahn, 2000). However, the concept of synchrony between humans and wearable robots is likely more complex than rhythmic coordination, since when a human wears a robot on their bodies, there is no “other” separate entity to observe, mimic, and synchronize with.

### Adaptation

A fundamental component of synchrony between actors is an individual’s adaptation in response to a partner’s behavior (Burgoon et al., 1993). Burgoon et al. (1995) argued that an actor’s tendencies to either reciprocate or compensate are affected by the individual’s needs, expectations, and preferences, called their interaction position (IP) in IAT (Burgoon et al., 1995; Burgoon et al., 2017). An interactant’s IP is comprised of three pre-interaction factors. The first is an individual’s *requirements*, or what they need during the interaction such as basic needs like comfort, or physical proximity (Toma, 2014; Burgoon et al., 2017). Second is the individual’s *expectations* about their communication partner and the exchange, for example, the verbal and nonverbal communication patterns that people anticipate from others based on what is typical for a given context (Burgoon, 2014). Thirdly, the individual has desires which are the communicator’s *goals* for the interaction (Burgoon et al., 1995; Burgoon, 2014; Burgoon et al., 2017). If the actor’s IP matches that of the receiver, IAT predicts the receiver will match, or reciprocate, the actor’s behaviors (Toma, 2014; Burgoon et al., 2017). This adaptation happens throughout the course of an interaction, and while there is an implication of intent when observing these interdependent patterns, Burgoon et al. (2017) point out that adjustments can be made automatically. In other words, reciprocity and compensation can be both conscious and subconscious (Burgoon et al., 1993; Guerrero and Burgoon, 1996).

Finally, another factor to consider in the adaptation process is the actor’s perception of the difference between their expectations and the actuality of their partner’s behaviors (Toma, 2014; Burgoon et al., 2017). Violations of expectancies are generally considered negative unless the person performing the violation is particularly rewarding (Burgoon et al., 1995). If their partner’s behavior is considered more positive than the actor expected, the individual will reciprocate with similar behavior. The difference between expectations and actual behaviors, (e.g., under- or over-reliance on automation), can have implications not only for adaptation, but also other factors related to collaboration, including trust and team performance (Lee and See, 2004; Nikolaidis et al., 2016).

### Barriers to synchrony

It is not always a given that humans will synchronize with another individual as there are several barriers that may prevent it. As synchrony can be either subconscious or unconscious, so too can the barriers to synchrony be intentional or not (Bernieri and Rosenthal, 1991; Burgoon et al., 1995; Fujiwara et al., 2022). Such challenges include factors related to intergroup differences, lack of understanding or comprehension between the interacting individuals, dislike or mistrust of one individual by the other, and contrasting goals or expectations for the interaction, (i.e., misaligned IP; Bernieri and Rosenthal, 1991; Burgoon et al., 1995).

Early evidence on human-machine synchrony indicates that barriers may be similar to human-human interactions (Lorenz et al., 2013). Kirkwood et al. (2021) identified several potential barriers to human-exoskeleton synchrony. They point out that as the exoskeleton is a *wearable* technology, users could have physical and psychological reactions to the machine on their body—like an invasion of personal space. Additionally, factors such as identification, understanding of the technology, perceptions and attitudes toward new technologies, and cultural norms in the work environment could all influence a user’s synchrony with the exoskeleton (Kirkwood et al., 2021).

Synchrony is particularly important for humans working with powered exoskeletons as the human and machine need to coordinate to become one entity (Kirkwood et al., 2021). While some dyads may be able to synchronize in one or two subcomponents, the challenge for synchronizing with a wearable robot, is that must be achieved across all three domains: body movements, rhythm, and timing. The interface between a human and exoskeleton is physical and influenced by the movements of the wearer and exoskeleton. Rhythm and timing are key to ensuring that the human and machine are not “opposing” each other, or acting out of phase, and can coordinate effectively to achieve the task shared-goal. Sensors in the suit can detect motion and send information to the robot controller, which then triggers a mechanical response in the machine to anticipate and match that of the human motion (Ronsse et al., 2011). However, this technology is still developing and there are many challenges to creating full-body, wearable robots, namely, humans vary not only physically, such as in height, strength, range of motion, etc., but also in learning and adaptability (Ronsse et al., 2011; Sposito et al., 2020). Since the action of one agent (human or wearable robot) can directly influence the action of the other, and the relative mechanical power of the robot (compared to the human) can be adjustable, sensemaking and agency are identified as two essential factors affecting synchrony in this context, and described further.

### Sensemaking

Sensemaking is an important concept related to adaptation and synchrony, as experiencing a new technology or feature can produce uncertainty and trigger sensemaking in order for the user to adapt (Griffith, 1999; Henfridsson, 1999). According to Weick et al. (2005), Sensemaking is a continuous, retrospective process in which circumstances are interpreted, and then action is taken based on that interpretation. The underlying assumption is that most people are operating within a mental script—not directly thinking about actions or actively interpreting meaning until something comes along out of the ordinary (Weick, 1993; Weick et al., 2005). When faced with a novel situation, individuals often use previous experience to interpret and react. For example, Griffith (1999) explored how members of an organization made sense of a new information technology (IT). During the initial release phase, members experienced high levels of ambiguity as to the meaning of the new technology in their work context, (e.g., role changes). Over time, as workers became more experienced with the technology, they used sensemaking strategies to adapt and create new organizational routines.

Within the domain of HRI, the concept of sensemaking has been utilized in two significant ways: First, from a robot interface-design perspective, there is a rich tradition within the human factors community of exploring how humans form mental models (internal representations or frameworks to understand and predict phenomena or action-consequences) of intelligent robots, and the factors that influence mental model formation (e.g., Kiesler and Goetz, 2002; Phillips et al., 2011; Tabrez et al., 2020). This work is very similar in conceptualization to sensemaking, in that humans are known to initially form primitive and incomplete, but functional, mental models of unfamiliar technologies that will continually improve with increased interaction and adaptations (Norman, 1983). This body of work also highlights that a robot’s physical appearance and language abilities may significantly influence the accuracy of mental models (Lee et al., 2005; Walters et al., 2008) leading to a human’s under- or over-estimation of robot capabilities. In the case of wearable robots, while the robot is not being seen by individuals during the interactions (as they are being worn), whether humans still anthropomorphize the robot/exoskeleton due to its intelligence and adaptive controls, and what design features affect sensemaking and mental model formation are important and intriguing questions to address.

Second, from a robot algorithm-design perspective, explainable AI techniques have focused on describing agent reward functions effectively, to enhance human-robot collaboration and task performance (e.g., Sanneman and Shah, 2022). These have been studied from the perspective of enhancing a human’s situational awareness (e.g., Sanneman and Shah, 2022) while not excessively increasing their mental workload; and included exploring the effects of different information types (Amir and Amir, 2018; Huang et al., 2019) and levels of information complexity Sanneman and Shah, 2022). While this body of work can be applied to the human-wearable robot context as such, it primarily stems from controlling highly complex industrial robots. Whether any such overt or explicit “explanation” of robot behaviors are necessary for sensemaking during training or operations of exoskeletons, is currently unknown.

### Agency

In general, scholarship has broadly defined the term agency as the “capacity to reflect, adapt, and act” (Gibbs et al., 2021, p. 156). While this definition implies actual capability, it is important to recognize that humans are not always aware of the bounds of their agency. Thus, we incorporate a perception-based alternative *sense of agency* which is considered as, “the feeling that one is in control over one’s actions and their consequences” (Ciardo et al., 2020, p. 2). Additionally, individuals not only perceive their own agency, but also will attribute agency to others which influences outcomes such as perspective taking (Ciardo et al., 2020). This sense of agency is critical for individuals to understand relationships with their environment and others around them, and in turn, it impacts interactions with another individuals (Kawabe, 2013; Lorenz et al., 2013; Fujiwara and Daibo, 2016). The increasing ability of intelligent machines to both appear humanlike while taking on human roles, such as decision-making, has brought the question of agency front and center to discussions on human-machine interaction (Edwards and Edwards, 2017; Sundar, 2020). Rammert (2008) argued that machine agency lies on a continuum of passive to active technologies and depends on the kinds of autonomous actions the human versus the machine can make. At the lower end of the spectrum are passive tools such as hammers that are operated completely by a human user are less agentic; then semi-active machines that are able to have some self-driven actions after human input, such as a standard vacuum; reactive machines such as those with sensors like washing machines or thermostat-based climate control; proactive machines that self-activate such as a simple customer service chatbot; and cooperative machines such as mobile robots or smart homes that may have equal or more agency than a human user (Rammert, 2008; Sundar, 2020). For users, their sense of agency and their attribution of agency to a machine counterpart can have impacts on decision-making and performance outcomes (Ciardo et al., 2020; Kirkwood et al., 2021). For example, Ciardo et al. (2020) found that humans experience a reduction in their own sense of agency when interacting with robots they perceived as having more agency than passive mechanical devices (such as air pumps).

Previous scholarship on humans and technologies have taken either an anthropocentric or a technocentric perspective, primarily focusing on either the human agent or the machine agent as the source of change or site of impact. Wearable technologies, however, provide a unique opportunity for a middle-ground perspective that looks at both humans and machines as mutually adapting influential agents (Guzman, 2020). To our knowledge, there is very little research exploring agency in the context of collaboration and adaptation between humans and wearable robots. As exoskeletons are a relatively new technology, we are interested in how users make sense of their interactions with the machines when exposed to a complex full-body, powered exoskeleton, and explore the concepts of agency and adaptation as they relate to human-machine synchrony. Thus, we pose the following research question:

RQ: How do the concepts of agency and adaptation manifest in human-exoskeleton synchrony, particularly as human users make sense of operating a complex whole-body powered exoskeleton?

To answer this question, we conducted qualitative interviews with novice and experienced users of an exoskeleton prototype, to gain a deeper understanding of their experiences.

## Methods

### Participants

This study used a convenience sample of 13 healthy male participants (5 experts, referred to below as “EXP#,” 8 novices, referred to below as “N#S#” to indicate session number as novices participated in multiple sessions whereas experts only participated in one session). None of the novices had used the exoskeleton prior to the study, while the “experts” were engineers with extensive experience in testing and operating the exoskeleton through its developmental phases (>3 months). Respective mean (SD) stature, body mass, and age were 1.8 (0.04) m, 84.4 (6.8) kg, and 36.8 (15.4) years for the novices; 1.8 (0.03) m, 83.9 (8.2) kg, and 31.2 (7.8) years for the experts. All participants performed short bouts (2-3 trials) of level walking, stepping up over a small obstacle, push/pull, load carriage, stationary load handling, and reach and point tasks. Data from the multiple sessions of the novices and the experts were pooled together and analyzed, to draw out the thematic elements. We did not use the data to try to differentiate between experts and novices or between the multiple sessions of the novices, owing to differences in sample sizes and imbalance in the data. Hence, the question addressed by the paper is what a user’s experience of the exoskeleton is, irrespective of whether they were a novice user, or had had some time to get used to the device. Prior to any data collection, written informed consent was obtained from all participants following procedures approved by the lead University’s Institutional Review Board (IRB).

### Description of exoskeleton and tasks

**FIGURE 1:**
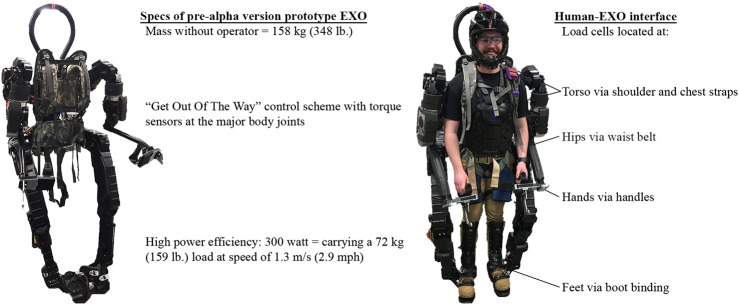
Pre-alpha prototype of the occupational whole-body powered exoskeleton (EXO) tested (Guardian^®^ XO^®^, Sarcos Robotics, www.sarcos.com). The red circled areas denote the human-EXO load cell (6-DOF force-moment sensor) interfaces where the EXO measures human-EXO interaction forces.

For this study, we used an early research-prototype version of the Guardian^®^ XO^®^ (EXO for brevity) developed by Sarcos Robotics, which is specifically designed for occupational applications (Figure 1). The EXO has a mass of 158 kg and includes 18 active degrees of freedom (DOFs) spanning the shoulders (flexion/extension, abduction/adduction, and internal/external rotation), elbows (flexion/extension), trunk (axial rotation and lateral bending), hips (flexion/extension, abduction/adduction, and axial rotation), and knees (flexion/extension). At the time of assessment, the EXO was still in the development stage, and the calf and ankle joints were not actuated. The EXO uses a patented “Get-Out-Of-The-Way” control scheme with torque sensors at the major body joints that allow the EXO to follow the human movement and amplify human joint torques (Jacobsen et al., 2014). With this technology, users can be aided to freely lift and handle loads up to 90 kg. To understand user movement intent, user input is obtained from embedded 6-DOF force-moment load cells located at the hands, feet, torso, and pelvis locations of the EXO. The EXO also has several tunable parameters, including virtual center of mass, gravity compensation, and actuation gains (magnitude of torque amplification).

Expert participants participated in one 2.5-h session and novice participants performed five 2.5-h sessions of simulated tasks, namely, walking, lifting, and transporting objects, pushing a cart, force-reproduction, and repetitive target-tapping while wearing the exoskeleton (See Park et al., 2023 for a more detailed explanation of study design and tasks). The sessions took place on separate days and the multiple sessions spanned across two to 3 weeks. Semi-structured interviews were conducted after each session.

### Interview protocol

After each study session, participants accompanied one of the experimenters to a separate location, where the interviews took place. Care was taken to maximize participant comfort and encourage unbiased responses, and their responses have been anonymized in this report. Interviews began with general questions, e.g., “how did today’s session go?,” followed by open-ended questions about participant comfort and confidence in using the exoskeleton, the physical- and cognitive strategies that participants used to learn and operate the exoskeleton, and their general perceptions of safety (Supplementary Appendix SA). Questions were similar between successive interview sessions, with minor modifications to suit the specific physical tasks performed on each day. Upon completing the interview, participants were asked for any final comments, and subsequently thanked for their time and escorted out of the interview location. Each interview typically lasted ∼30–40 min. The interviews were then transcribed for analysis.

### Coding protocols/methods

In this project, researchers used an iterative approach where prior research on human-machine synchrony and sensemaking guided the coding process while also looking for emergent codes and themes (Tracy, 2020). After interview data were transcribed, the transcripts were uploaded in the computer-assisted qualitative data analysis software Atlas.ti for coding and analysis. After reading each transcript, researchers conducted line by line analysis of *in vivo* coding (Tracy, 2020), which consists of using the participants’ own language to create codes, to discover how participants achieved synchrony while wearing the exoskeleton and how they made sense of those experiences (Strauss and Corbin, 1990; Lindlof and Taylor, 2019). After *in vivo* coding, similar codes were grouped together and the constant comparative method was used to conduct as many rounds of axial coding as possible, or grouping codes into categories and subcategories, that were necessary to identify emergent themes (Lindlof and Taylor, 2019). After two rounds of axial coding, the emergent themes were identified as findings.

## Findings

**FIGURE 2:**
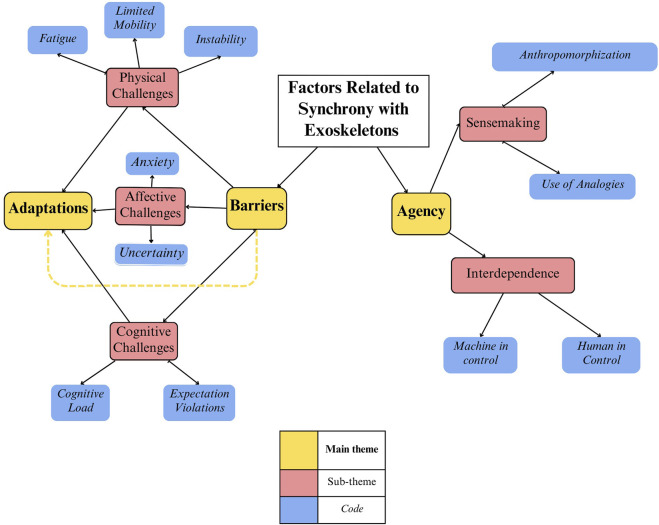
Thematic analysis results.

Our findings revealed that wearers of the exoskeleton faced challenges to the process of synchrony for which they needed to adapt in order to perform tasks. Additionally, participants used sensemaking strategies and contemplated their sense of agency as related to the EXO in order to understand their role in the human-machine partnership (Figure 2). We unpack these two main themes and their subthemes below.

### Synchrony

We found that participants faced a variety of challenges that required adaptation in order to synchronize with the exoskeleton and perform the given tasks. Challenges of synchronizing with the technology fell into three major themes: physical, cognitive, and affective. Physical challenges included fatigue, tension, jerky movements, and limited mobility in the exoskeleton. Affective challenges included expectancy violations while wearing the suit, uncertainty of how the suit was moving while using it, and feelings of fear and intimidation. Lastly, cognitive challenges included the nuanced ways that concentration and mental energy impacted suit use. We found that participants developed and used strategies for synchronizing with the exoskeleton and performing the given tasks that were thematically related to the respective physical, cognitive, or affective challenges. Thus, there is a relationship between the barriers faced and the adaptations needed to address those barriers.

#### Physical challenges

The physical challenges that participants described while wearing the exoskeleton included increased levels of fatigue, feelings of instability, and limited mobility.

##### Fatigue

One participant stated, “everything is tense and it, it fatigues out because it just takes longer to do the action, so you’re, you’re all tensed up and for way longer.” (N8S2) Another participant explained that certain parts of the body are more susceptible to these challenges when he said, “exhausting on my back, you know, like, um, the feet—being in the EXO, my feet kill me, and when I’m not in the EXO, they’re fine.” (N4S3) Participants tried to make sense of why they got so fatigued while using the exoskeleton by saying: “Just trying to figure out what was going on with the robot and it felt so weird and, um, by the time we got … things dialed in I was getting tired” (N3S3). Additionally, one participant tried to describe the discomfort that led to fatigue: “I just feel like—it’s not even so much that it’s, like, muscularly tiring, it’s, like, it just hurts your body, kind of. That’s how I feel, at least, it’s … weird, uh, I mean, and it’s probably 'cause it's working muscles that wouldn’t otherwise be worked.” (N8S5) It was clear from participants’ comments that strength and muscle conditioning were needed to build stamina for regular exoskeleton use. Additionally, while the exoskeleton made human wearers stronger in some areas, there was the cost of increased exertion to perform basic movements.

##### Instability

Participants also reported feeling unstable or concerned about making movements that would put them off-balance, which would have implications for their ability to perform tasks. For example, one commented, “it just doesn’t feel balanced, so you can’t make these big steps to move.” (N4S1). Another user noted the difficulty in just maintaining stability, “it’s hard to balance with the robot especially because it’s pulling on different points of you. So you have to have—like your whole body balanced.” (EXP2). Other users described how quickly their movements in the suit would throw them out of balance necessitating careful, deliberate movements, “Turning around, I had to really pay attention to what I was doing, and keeping the balance, I had to pay attention to what I was doing” (N6S1). Although the exoskeleton’s mass did not need to be supported by the users, the exoskeleton’s inertia was still perceived by the user, and since the device was not self-balancing, the users were responsible were balancing the human-exoskeleton system. However, users’ perceptions of their (in) ability to balance the suit impacted their movement decisions and led to performance challenges. As one participant noted after watching others, “people try to walk to a point where they’re, you know, too quick for the exo to move or they’re not recognizing the feel of the exo with their own body. So they’ll—they’ll get out of balance and they’ll stumble forward or they’ll have very heavy footsteps” (EXP4). This seeming lack of control over stability is one of the key physical barriers interfering with developing synchrony with the machine.

##### Limited mobility

The final physical challenge that participants described was feelings of tightness and limited mobility while wearing the exoskeleton. “It definitely limits your motion in ways.” (EXP1). One participant described this challenge in-depth, “But in the Exo, it’s like I have to be in a very, very, very tight—like it’s—the parameters of where you can take the step and stand up are—are much tighter.” (N4S2) Many participants echoed that they were unable to stand up straight while the exoskeleton and instead had to assume a crouching posture to use the suit. Another participant explained how mobility challenges also included the speed in which the wearer can move by saying, “it slows you down but it allows you to carry more, it’s kind of a tradeoff there.” (N2S4) This shows that some participants were willing to adapt their movements in order to achieve a goal.

##### Physical adaptations

To address physical challenges, participants had to change their natural body movements in order to perform tasks and maintain balance. In particular, users adjusted the way they walked, describing it as, “this like penguin thing where you’re shuffling 180°” (N4S1), or “more of a—a tip-toe motion to put your foot down flat, uh, when walking to make it not be so clunky.” (N3S1). One participant noted how he, “tried to keep the arms as close to the exo body as possible and then try to stay as centered as I could” (N6S5). Overall, participants experienced a tradeoff of increased physical strength, for decreased stability, mobility, and energy efficiency.

#### Cognitive challenges

The second salient challenge participants faced were cognitive challenges while wearing the exoskeleton. Cognitive challenges were categorized into two types: cognitive load and violations of expectations.

##### Cognitive load

One participant described the higher levels of mental energy required to do simple things in the exoskeleton such as walking. This participant stated, “I think in the exoskeleton, because walking back and forth takes, I’ve got the robot around me, it takes a bit more thinking about it.” (N5S3) Another participant echoed this sentiment for a different task saying they, “Have to really pay attention to try.[to] hit the tape target.” (N6S4) Though the tasks of walking and hitting a target would not require a great deal of effort for able-bodied individuals, the addition of the exoskeleton required participants to actively think about what they were doing in order to accomplish their goal.

The higher cost of mental energy while using the exoskeleton was also present in more complicated functions including lifting a heavy item from a low shelf. One participant stated, “getting down low enough, um, to pick those weights off the lowest shelf still required some thought.” (N7S1) Lastly, one participant indicated that it might not be possible to turn off the awareness of being in the suit: “it’s night and day. I mean, you walk, you know, without even thinking about it. You breathe without thinking about it. You blink without thinking about it. So I mean, this is something like you have a 400-pound thing on you, you know? You—you think about it all the time when you’re in it.” (N4S3).

##### Expectation violations

Participants described a feeling of disorientation when using the exoskeleton did not match their prior expectations of the technology. One participant stated:

“I expected it to feel more like a sort of weight on you as the operator and the fact that it doesn’t, the fact that it feels like it’s suspended in air basically was interesting. That was – I mean, I guess I should have predicted it would feel like that. But it was an odd sensation.” (N5S1)

This participant seemed to have difficulty reconciling his expectations from seeing the large exoskeleton, and the feeling of actually wearing it. Another participant echoed the sense of unusual and unexpected sensations, stating, “with the exoskeleton you don’t have to push with your arms, which was kind of freaky.” (N6S3) Overall, these quotes exemplify that it was difficult for participants to have realistic expectations before having hands-on experience with the suit.

##### Cognitive adaptations

Participants described the increased cognitive effort it took to perform tasks in the exoskeleton, and some participants explained that thinking could actually be counterproductive. “I was able to smooth it out by just simply not thinking about it. I felt like when I would screw up it was because I was overthinking it rather than just doing it.” (N3S4) One participant suggested trying to focus on *not* focusing: “I tried to focus on being more natural, particularly with the walking, on not sort of thinking about what I’m doing so much but just trying to walk as, as if I would normally.” (N5S3) Participants attributed successful coordination with the exoskeleton with reduced concentration. “You can’t think about it too hard.’cause then your brain gets in the way [of balancing].” (N2S2).

#### Affective challenges

In addition to physical and cognitive challenges while using the exoskeleton, participants described affective challenges. Affective challenges included uncertainty about how the suit would react during use, and feelings of fear while using the exoskeleton.

##### Uncertainty

Another subtheme that emerged from affective challenges was that participants were uncertain of how the suit was functioning and what it would do when they initiated movements. For example, one participant stated, “it’s hard to have a good, uh, proprioceptive feel of exactly what the robot’s doing all the time.” (EXP2) This participant suggested that the exoskeleton should have more feedback mechanisms to help the wearer understand what is happening when they use the suit.

Another participant explained that since the exoskeleton takes the brunt of physically demanding tasks, it is difficult to know when it stops applying force. When reflecting on a task they completed with the exoskeleton, one participant stated:

I couldn’t even tell I was pushing on it, like, I had to look and I was, like, “Oh, yeah, I’m still pushing on it. Like, am I applying force?” And they were, like, “Go up and down,” like, “Oh, yeah, it's still there.” So, if, if you’re not, like, actively moving it, I feel like you don't feel anything. (N8S5).

This participant mentioned how uncertainty of what the exoskeleton limbs are doing at all times could create problems for users and other potential safety challenges. Another participant explained how uncertainty of exoskeleton movements can disrupt suit functionality when he stated that wearers, “don’t recognize what the robot’s doing around them and so, they get into positions that they’re unable to recover from” (EXP4).

##### Anxiety

The last subtheme related to affective challenges of exoskeleton wearers was feelings of fear or intimidation that made it difficult to synchronize movements with the suit and complete tasks. One expert noted the recursive interaction of performance challenges and anxiety, “they are moving faster than the robot’s able to respond or they’re very apprehensive and nervous so they are very stiff” (EXP4). The major source of anxiety was the participants not knowing what might happen if they moved incorrectly. When operating the suit, users, “had to be on guard all the time because you never know what was gonna happen” (N6S2). One participant described the anxiety they have of potentially falling while wearing the exoskeleton. “I don't wanna fall in a weird way” (N8S3). Even after a fall recovery, participants experienced apprehension, “when I fell backwards off the–the force plate, like that was just ‘cause I’m still not used to it pulling me back … you gotta get much more forward which is a little scary” (EXP1).

##### Affective adaptations

Much like the cognitive adaptations, participants discussed the need to let go of tension and fear in order to perform tasks in the exoskeleton. “That’s probably the biggest thing is overcoming apprehension and relaxing when–when they’re attempting to do something.” (EXP4) The majority of users felt relaxation was the key to successfully working in the suit. “I think, becoming more relaxed is a big part of it” (N7S4), or, “I was too tense yesterday. And I think that I was better at it today. Still not near as good as without the exo.” (N5S5) This relaxation seemed to increase with time spent in the suit. One person processed the reasons behind their improved performance, and proposed the relaxation came from learning the suit, “After relaxing a little bit and, um, just having the opportunity to get used to the—the robot.” (N3S1).

### Agency

The second major theme that was revealed in our analysis was how participants perceived their own agency as relative to that of the exoskeleton. Within the topic of agency, we found two subthemes: participants engaged in sensemaking to understand the exoskeleton technology; and participants had a preconceived idea of interdependence and who should be in control-the human or the exoskeleton.

#### Sensemaking

Our findings indicated that participants engaged in sensemaking that involved the use of analogies or anthropomorphizing the exoskeleton. Participants used a variety of tactics to understand the novel experience of wearing and performing tasks with the exoskeleton. Two main themes emerged from the ways that participants made sense of using the exoskeleton including analogies to other tools or equipment and anthropomorphizing the exoskeleton.

##### Exoskeleton analogies

When describing tools or equipment that reminded them of their experience with the exoskeleton, participants mentioned riding a bike, playing an instrument, using a forklift, or operating a car. One participant described their experience using the exoskeleton as similar to, “Wearing a big hiking backpack, um, that is somewhat similar ‘cause you’re wearing the, um, big thing and there is a mass on your back that you have to manipulate.” (N2S4) Another participant described the experience as similar to riding a motorcycle when he stated, “I think operating motorcycles is just another example of being relaxed while, um, moving a heavy machine and trying to get to the point where … you’re one with the machine.” (N7S5).

Analogies participants used to describe the exoskeleton ranged from positive to neutral to negative. One participant seemed to feel trapped, stating, “I feel like it would be more like a prison. Like, that’s what you go make the inmates wear, and they go lift rock outside.” (N8S5) Another participant described the process of adapting to a different way of moving in the exoskeleton when he stated, “I’m trying to—I really reg—regulate how exactly the form of my gait like how my feet move and everything like that and I’ve done that a lot in like marching band.” (EXP2) One participant described doing tasks in the suits as feeling like a, “95-year-old man that has the power of Superman” (N4S2).

Lastly, some participants explained that using the exoskeleton was not analogous to any other experience they have had before. One participant explained, “It’s, uh, it’s completely different experience I think.” (EXP3) Another participant stated, “I’ve operated a lot of machinery, uh, throughout my life and I’ve never operated anything like that.” (N3S3) These quotes showcased that while most users have some experiences to frame their experience with the exoskeleton, others found the experience completely new and unique.

##### Anthropomorphization

Another emerging theme was that participants also made sense of using the exoskeleton through anthropomorphizing the suit. In these instances, participants tended to describe the exoskeleton as a separate robotic entity that they needed to work with to complete tasks effectively. When learning how to use the exoskeleton, one participant stated that using the suit becomes, “more smooth as you kind of learn what the robot likes and what it doesn’t like.” (N2S5) In this quote it was interesting that the participant described the exoskeleton as a robot that would have emotional preferences for how the wearer should move.

Some participants explained that using the exoskeleton is less about accommodating its preferences and more about combating it. One participant explained: “I have found the best way to combat that is to kind of anticipate it and—and fight it. Fighting is a loaded word, but like that’s what you’re doing.” (EXP1).

While some participants described the exoskeleton as a robot that needed to be fought into submission, other participants mentioned how fighting the exoskeleton was counterproductive. One participant stated, “if you’re out of sync and you’re fighting it the more you fight it the more you’ll become out of sync and the less the controller will respond.” (EXP4) These quotes exemplified that anthropomorphizing the exoskeleton resulted in different perspectives of the technology and different approaches to using the suit effectively.

Lastly, some participants talked about the exoskeleton as though it were a fellow human capable of having emotions, and even expressing sentiment about the machine itself. For example, one participant stated, “The robot kinda acts, and, uh, it’s—it's just—you start to—you know, it just gets tired.” (N4S1) This participant additionally described thinking of the exoskeleton as a human, “You basically have another human being on your body that you’re controlling.” (N4S1) One user described his time watching other participants interact with the exoskeleton much like watching a child develop, stating, “I was with the robot a lot while it was being brought up.” (EXP3) This quote in the context of talking about his designing the suit’s internal electronics, then seeing the full suit in action, seems to indicate an underlying parental sentiment toward the exoskeleton. Another participant expressed disappointment with his relationship to the machine commenting, “…if I had the opportunity to just work with it [the exoskeleton] by myself … walk around, pick things up. You know, just concentrate on me and the exo.” He lamented that concentrating on the tasks, “took away me trying to bond with the exo.” (N6S2) This indicates the participant wanted more than just a physical connection to the machine, but perhaps an emotional connection as well.

#### Interdependence and control

Our results suggest that users coordinate and attempt to synchronize with the exoskeleton to perform tasks through understanding their interdependence with the suit. One factor that influenced the management of these dependencies was the attribution of control to either the human user or the suit. This in turn, would correspond to which actor—the human or the machine—should do the adapting in order to synchronize and accomplish a task.

##### Human dependent on the machine

Some participants described their actions as being determined by the needs of the exoskeleton: “it doesn’t want to—uh, it just doesn't feel balanced, so you can’t make these big steps to move—like to rotate, you know?” (N4S2) In this case, the exoskeleton was limiting the physical mobility of the wearer by failing to maintain balance that would enable a user to achieve a full range of natural movements. To move, the human user had to adjust the way they walked: “I think it was more of, um, like they said in the beginning just walk forward and pull the robot from your waist. Um, which I still have trouble doing it occasionally.” (N2S5) Additionally, one user mentioned needing to adjust their movements in various ways to help increase performance. “If I’m walking and it’s got a load on my back, I can straighten my back out to help the system perform better from–from walking, for example.” (EXP4).

When a participant discussed performing well with the Exo, they attributed it to their learning improvements, or improving adaptation to the suit. “I was a lot better at it because I learned to relax, and I wasn’t–when I was tense, the robot would go too fast, and then you had to counter that.” (N6S4) Another participant stated, “it was more me being clunky because I wasn’t used to, um, the different posture changes that I had to have, and the mass of the machine.” (N3S1) These users seemed to place the ownership of (un) successful performance in the suit on the human wearer. In other words, humans should adapt to meet the functioning of the exoskeleton rather than the machine adapting to human movements.

##### The machine should adapt to the human

Other participants believed the exoskeleton should respond to human movements: “So I know that it’s not gonna fight me in any way, it’s not gonna try to follow any predetermined motion. It’s going to do everything it can to follow my hand because that’s how it’s built.” (N2S5) Additionally, challenges to coordination we perceived by some users as being the fault of the suit not performing as it should. “It felt like sometimes I’d be pulled or shoved in a direction that I, I didn't feel like I caused.” (N8S1).

One participant attributed coordination challenges to the unpredictable behavior of the exoskeleton. “The thing is you don’t know how it’s really gonna act, especially like the legs, and like you kinda do, but you have an idea, but you’re never 100 percent sure of what exactly it’s going to do.” (N4S3) Another participant stated that changes to the human-machine connection points would alleviate challenges of synchrony: “with a more flexible harness and things like that you might be able to get more intuition of that” (EXP1). Rather than considering adaptation to be solely the responsibility of the human wearer, these participants appeared to consider the exoskeleton as a machine that needed to change in order to adapt to the human.

Overall, the participants were divided on the issues of control and responsibility. When considering whether the human or the machine should lead, some participants described needing to adapt to fit the movements of the exoskeleton, while others indicated the need for the exoskeleton to adapt and better follow the lead of the human wearer. They also differed on the attribution of responsibility for challenges or faults; with some participants pointing to the exoskeleton, and others taking the blame themselves for not adjusting to the exoskeleton. Opinions on these issues, however, did not remain static as a few participants expressed feeling in control of the robot in one session, “I know I have enough control of the robot that anything I do the robot will do.” (N2S3), and letting the exoskeleton take control in a different session, “I’m just walking and the exo will take care of the rest of it.” (N2S5).

## Discussion

As machines become more advanced, taking on more responsibilities over work and actions, relationships between humans and machines are becoming increasingly interdependent. Humans are no longer autonomous agents simply using tools, we must share decision-making and yield some agency to machine counterparts. One rapidly growing area of research is wearable robots called exoskeletons, which are designed to augment human strength and stamina, while maintaining the human abilities of creativity and adaptability. In order to accomplish tasks, the human user and exoskeleton must cooperate to perform as one unit. Thus, the learning process in this case can be framed as human users finding the optimal way to interact with the machine in order to get the best performance, rather than the human necessarily needing to adapt to the machine. A study by Tyagi et al. (2023) however reported that even using a passive arm-support exoskeleton involved greater motor coordination and planning efforts, compared to the control condition (i.e., no exoskeleton use). This suggests the use of an exoskeleton involves different neuroadaptation strategies than other types of technologies. In other words, the human may use a variety of adaptation methods to synchronize with an EXO and share control over one body. Thus, our findings are consistent with, and expound on previous literature studying human-exoskeleton interaction.

Though synchrony is generally understood as being a fundamental process in human behavior, there are several barriers that can disrupt individuals from fully synchronizing with another agent. This study contributes to the human-machine interaction literature by exploring the process of, and barriers to, synchrony between humans and wearable, machine agents. We were guided by the overarching question asking how agency and adaptation manifest in human-machine synchrony as human users make sense of a complex whole-body powered exoskeleton. We found that participants in our study engaged in sensemaking to both understand and address the challenges they faced while trying to accomplish tasks in a whole-body powered exoskeleton. A thematic analysis revealed that users faced three main types of challenges: physical, cognitive, and affective. The findings suggest that users were adapting to the exoskeleton, attempting to synchronize their movements to that of the exoskeleton in order to accomplish tasks. Adaptations were related to the particular type of challenge users faced, whether they were cognitive, affective, or physical. Physical challenges including fatigue, instability, and limited range of motion, caused participants to change their natural body movements, to adapt to the exoskeleton. To adapt to cognitive challenges, participants had to change the way they thought about using the exoskeleton and reconcile expectation violations. Lastly participants experienced affective challenges including anxiety and uncertainty, and had to consciously relax and allow for more time in the machine in order to adjust and synchronize.

Decades of empirical work has shown that humans naturally want to synchronize with others, including machines (Burgoon et al., 2014; Lehmann et al., 2015; Fujiwara et al., 2022). Our study provides further evidence of this process in human-machine interactions, specifically, in human-exoskeleton relationships. While there is early research exploring human-machine synchrony, its antecedents and outcomes (Fujiwara et al., 2022), this is the first paper to look at interaction adaptation and synchrony that results in shared control over a human body. It is worthwhile to note that there are a few recent papers examining physical an/or cognitive adaptation with an exoskeleton (e.g., Park et al., 2023; Tyagi et al., 2023), however, these studies did not encompass a shared-control perspective. This study also shows that this shared control has implications for an individual’s assessment of agency, which aligns with previous research on perceptions of agency in human-machine relationships (Ciardo et al., 2020).

As full-bodied, powered exoskeletons for broad use are still early in the development stages, this research is among the first to explore how users make sense of a new and unique technology. Our findings indicate that participants used sensemaking strategies to understand, and react to, changes in their senses of agency as relative to the agency of the exoskeleton. Specifically, participants engaged in sensemaking such as using analogies and anthropomorphizing the exoskeleton in order to understand the machine itself, how to relate to it, and ultimately how to adapt and synchronize with it. Some participants compared wearing the exoskeleton to other types of wearable items such as backpacks and ski boots; others thought of the exoskeleton like operating a larger machine such as a motorcycle. Participants also anthropomorphized the exoskeleton, ascribing it agency as a separate entity from themselves. These findings are consistent with previous work in human-machine interaction showing that individuals use mental shortcuts, or mental models, to understand and set expectations for intelligent robots, which in turn guides their interactions (e.g., Kiesler and Goetz, 2002; Phillips et al., 2011; Tabrez et al., 2020). The different frameworks for understanding the exoskeleton likely played a role in how participants perceived their own agency as relative to the exoskeleton’s, and which agent–the human or the machine–should be in control of movement. Previous research has shown that humans experience a change in their understanding of their own agency when working with a partner, whether it be human or robot, however a more anthropomorphized robot can cause participants to experience a loss in their own sense of agency as they yield more agency and control to the robot counterpart (Ciardo et al., 2020). Further empirical work is needed to determine whether there is indeed a causal relationship between an individual’s understanding of an exoskeleton and the implications for their own sense of agency. For example, if a participant thought of the exoskeleton as a tool or wearable object, would they have a higher or lower sense of agency than an individual who anthropomorphizes the suit; and would this in turn influence how actively they tried to “control” or “adapt” to the machine.

### Practical implications

There are several practical implications for designers, managers, and users of powered exoskeletons. The sense of agency a human-user has with an intelligent machine, may guide practical decision-making in many situations: for example, i) if a performance error is anticipated, does the human user take ownership of the situation to correct their inputs to the machine or wait for the machine to sense/adaptively correct its outputs; and ii) if human expectations are violated in terms of task performance strategies by the machine, do they trust that the system knows better and acquiesce, or fight to regain control. These are issues that are likely to be determined by what agency the human user attributes to themselves and the machine. Knowing how the human-machine partnership impacts the human’s understanding of their own agency is thus critical for safety and performance when deployed in a work environment. If employees feel they are not fully in control of their own actions, or there is uncertainty regarding which entity is responsible for specific decisions, this could lead to challenges in operating the exoskeleton and an increased likelihood for accidents and further liability concerns. Additionally, reductions in agency can have a negative impact on employee’s job satisfaction (Feldman et al., 2018).

From a designer’s perspective, the ideal human-machine partnership in different scenarios and the agency human users “should” develop with the machine, should be carefully considered and communicated to the users of such technologies, along with training about the machine’s capabilities and limitations in a variety of scenarios. It may also be wise to consider how diverse users may make sense of the technology. Exoskeleton designers/control engineers can then guide sensemaking by how they introduce and train users to elicit more positive responses and help users maintain a higher sense of agency and reduce anxiety. For example, designers can ensure the users understand that while the suit itself may be more physically capable, the human is still in control. Lastly, designers can offer users a basic understanding of how the exoskeleton works and what it does from a mechanical and controls standpoint. In other words, making the user aware of the suit’s state and intention. Comparably, Stirling et al. (2020) suggested that an exoskeleton should enable the user to maintain sufficient cognitive abilities for processing task- and stimulus-related information while using the exoskeleton. When designing control interfaces, researchers need to not only consider what is the most efficient interaction between human and machine, but also what allows the human to maintain the highest sense of agency. This can be achieved by either creating a set of initial interactions that can allow a user to actively explore agency using their own sensemaking strategies, or intentionally program a series of technology interactions that can help create the expected sense of agency.

Our findings also reveal that although participants in our study did attempt to synchronize with the machine in order to accomplish tasks, the cost/benefit ratio, and the need for human adaptation is a barrier to synchrony and may reduce human trust, technology adoption and human-machine performance. It is also currently unclear as to how long participants would have continued to make adjustments to their natural body movements and their mental models of the interaction and expectations from it, to create a sufficient level of synchrony that can sustain the human-machine partnership. Previous studies have shown that individuals have a range of desire and ability to adapt to a robot counterpart (Nikolaidis et al., 2016). As such, it is possible that some users of exoskeleton technologies will be unwilling or unable to adapt to the machine’s movements or experience more challenges when attempting to adapt to this new technology.

## Conclusion

In summary, we have provided insights into how individuals make sense of new machines, not only designed to be partners or augment human abilities, but also be physically worn by the user. As users attempt to synchronize with the machine, an individual’s conception of their own agency as relative to the machine becomes salient. Understanding how agency plays a role in adaptation and synchrony is important for enabling the design of bidirectional coadaptation principles, and for the safe adoption of these new technologies.

## Data Availability

The datasets presented in this article are not readily available because some of the original data may contain identifiable information. Requests to access the datasets should be directed to jnwilkenfeld@ucsb.edu.
