# Supplementary material for: Sensemaking, adaptation and agency in human-exoskeleton synchrony
Source: Front Robot AI. 2023 Oct 12;10:1207052. doi: 10.3389/frobt.2023.1207052 (PMC10602643; doi:10.3389/frobt.2023.1207052)
Supplement: Supplementary file 1 [file DataSheet1.docx]

**Appendix A**

Exit Interview Questions - Expert User

1. What is your definition of being in-sync with the exoskeleton?
2. To what extent did you feel that the exoskeleton is in sync with your body?

Not at all Slightly Moderately Very Entirely

1. Did you feel, at any point, that the operation of the exoskeleton is analogous to using any other device/tool/machine or performing other kinds of physical tasks? Please elaborate.
2. Do you do any mental preparation or visualization immediately before starting a task?
3. Do you see a mismatch in how people expect the robot to move and feel prior to using it, vs how it actually moves and feels?
4. Do you think that doing any exercise/training outside of the exoskeleton would help/has helped you achieve better performance in the exo?
5. Do you have knowledge about, or can you guess about the control system and inner workings of the robot? Does this knowledge help you achieve the best performance out of it?
6. Do you think such knowledge would help a lay person/worker to learn the robot better?
7. Please tell us what you think about the exoskeleton fit and comfort, and how it restricts your body’s natural motion, using this scale (show them the scale below and ask these questions).


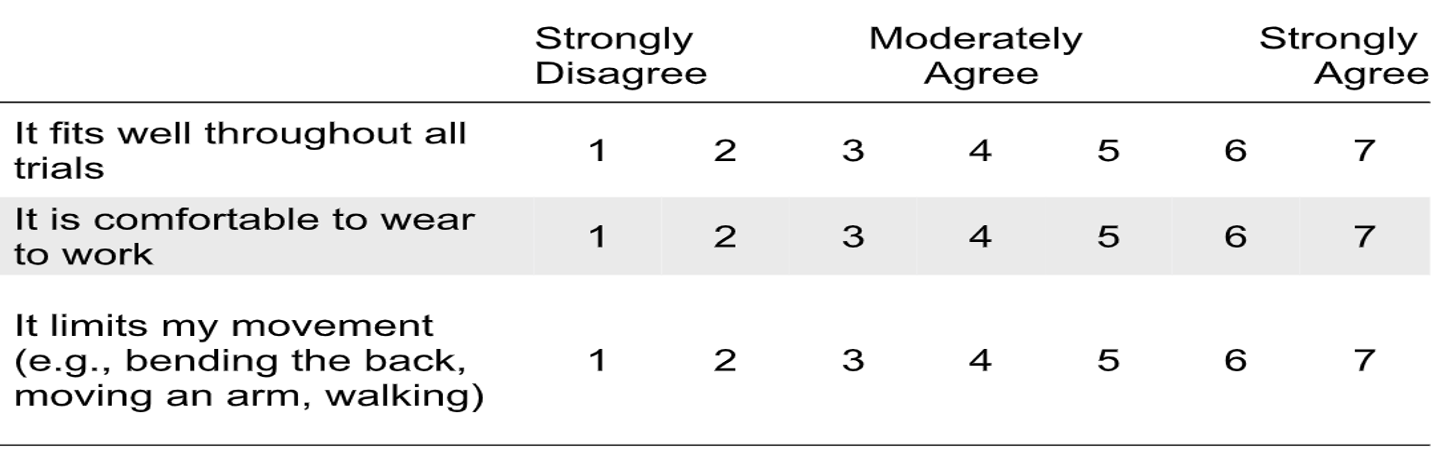


1. Compared to without the exoskeleton, how does the device affect the following characteristics of your work? *Cycle through all tasks: Walking, Cart pushing, Target tapping, Force control and Shelf lift*
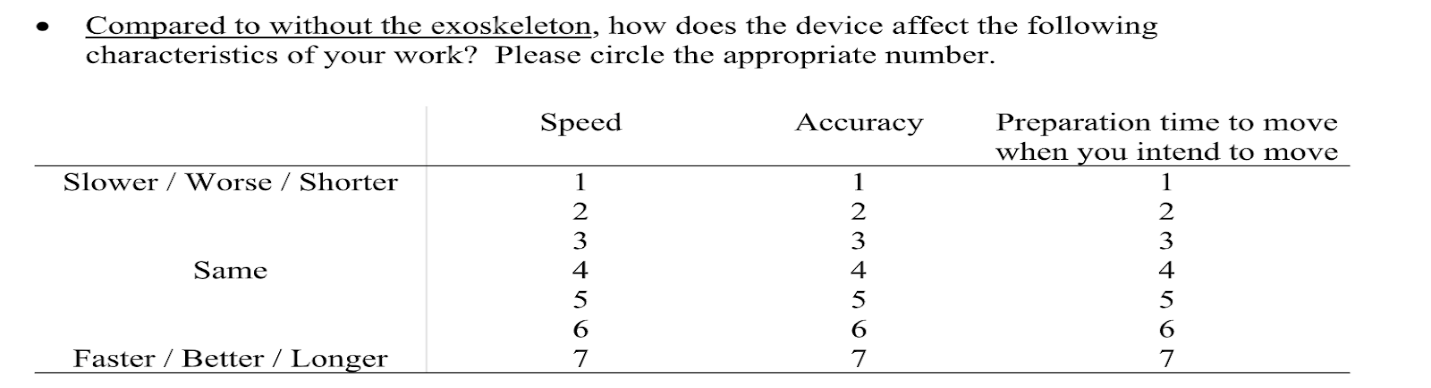

2. We are trying to understand which tasks the exo could be optimally applied to, to be useful. For each task, how helpful do you think the exoskeleton was, for performing it?


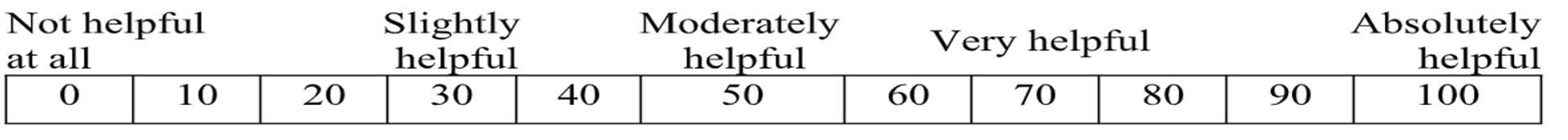


**Appendix B**

Exit Interview Questions - Novice Users

1. How natural did your movements feel in the exo, as compared to the no-exo condition?

| Very Natural | Natural | Average | Unnatural | Very Unnatural |
| --- | --- | --- | --- | --- |

*Probe: Were there any situations where the actual operation of the exoskeleton felt different from what you expected it to be? Please elaborate.*

1. On completing this session, how confident are you to use the exoskeleton to perform tasks?

| Very Confident | Confident | Moderate | Unconfident | Very Unconfident |
| --- | --- | --- | --- | --- |

*Probes: Task specific probing (Walking, Load carriage, Cart pushing, Step up/down, Target tapping, Force control and Shelf lift)*

1. How would you define being “in sync” with the exoskeleton?
2. To what extent did you feel that the exoskeleton was in sync with your body?

| Not at all | Slightly | Moderately | Very | Entirely |
| --- | --- | --- | --- | --- |

*Probes: What specific actions were the most/least intuitive? (E.g., bending forward, lifting arms etc.)*

1. How much mental effort did you have to exert during the tasks, as compared to the no-exo condition?

| Much Higher | Higher | The same | Lower | Much Lower |
| --- | --- | --- | --- | --- |

*Probes:* *Do task-specific.* *Can you describe the actions or movements that caused the most mental effort? Why do you think that happened (compared to not using the exo)? [E.g., maintaining forward/sideways balance, avoid hitting the shelves, maintaining a hold on the handles etc. – try to get them to be as specific as possible].*

1. How much physical effort did you have to exert in the exo condition compared to the no-exo condition?

| Much Higher | Higher | The same | Lower | Much lower |
| --- | --- | --- | --- | --- |

*Probes: Can you describe the actions or movements that caused the most physical effort? Why do you think that happened (compared to no exo)? Least/why?*

1. Please tell us what you think about the exoskeleton fit and comfort, and how it restricts your body’s natural motion, using this scale *(show them the scale below and ask these questions)*. *For Fit, talk about straps and joint alignment (between exo and human)*


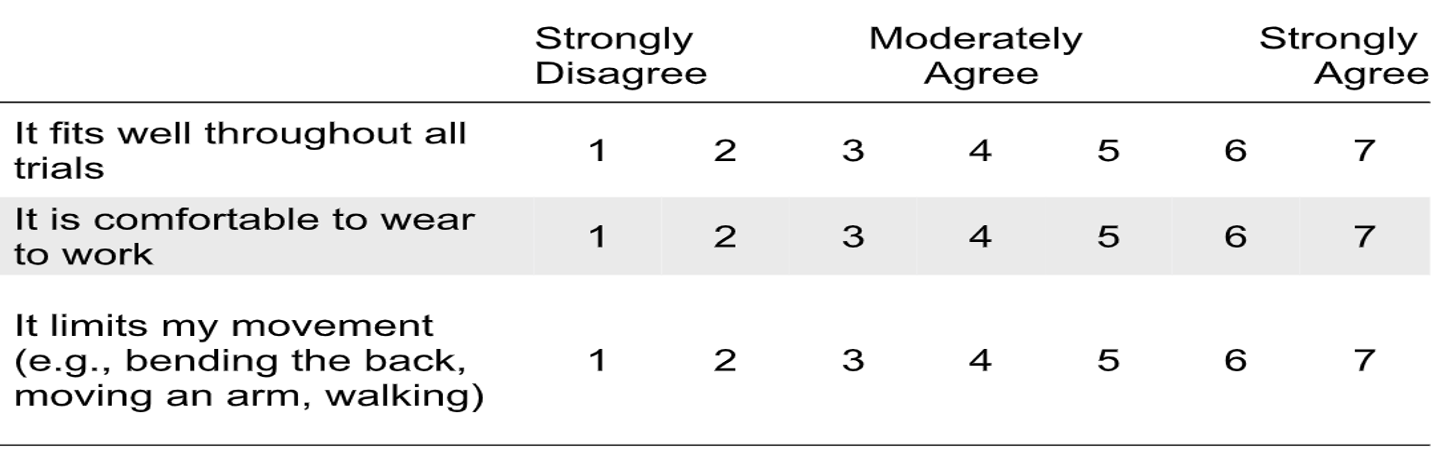


1. Compared to without the exoskeleton, how does the device affect the following characteristics of your work? *Cycle through all tasks: Walking, Cart pushing, Target tapping, Force control and Shelf lift*


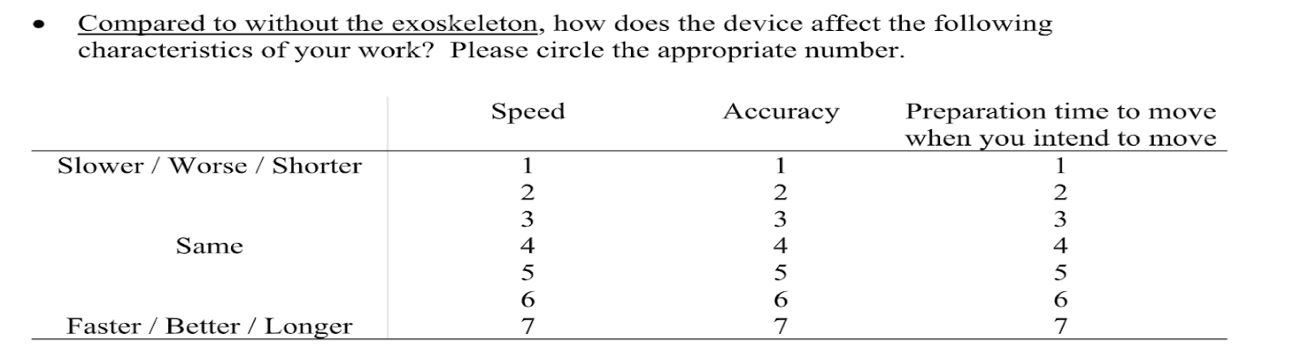


1. We are trying to understand which tasks the exo could be optimally applied to, to be useful. For each task, how helpful do you think the exoskeleton was, for performing it? (Load carriage, Cart pushing, Force control and Shelf lift)


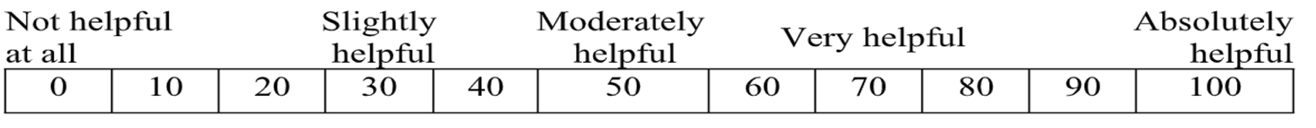


1. How safe did you feel in the exoskeleton (for yourself and the environment around you)?

*Probe: (why? elaborate)*

1. What aspects of training were most useful for learning to operate the exoskeleton?

*Probe: (why? elaborate)*

1. What aspects of training do you think could be further improved or emphasized more, to aid in operating the exoskeleton?

*Probe: (why? elaborate)*

1. In which tasks do you think you improved the most?
2. Anything else we missed, that you want to talk about?
